# Preoperative Cognitive Impairment as a Predictor of Postoperative Outcomes in Elderly Patients Undergoing Spinal Surgery for Degenerative Spinal Disease

**DOI:** 10.3390/jcm10071385

**Published:** 2021-03-30

**Authors:** Hyung Cheol Kim, Seong Bae An, Hyeongseok Jeon, Tae Woo Kim, Jae Keun Oh, Dong Ah Shin, Seong Yi, Keung Nyun Kim, Phil Hyu Lee, Suk Yun Kang, Yoon Ha

**Affiliations:** 1 Department of Neurosurgery, Spine and Spinal Cord Institute, Severance Hospital, Yonsei University College of Medicine, 50-1, Yonsei-ro, Seodaemun-gu, Seoul 03722, Korea; ns.kimhc@gmail.com (H.C.K.); anseongbae@gmail.com (S.B.A.); sisunsingum@naver.com (H.J.); cistern@yuhs.ac (D.A.S.); yiseong@yuhs.ac (S.Y.); kimkn@yuhs.ac (K.N.K.); 2 Department of Neurosurgery, Inje University Sanggye Paik Hospital, Inje University College of Medicine, Seoul 01757, Korea; xodn416@gmail.com; 3 Department of Neurology, Severance Hospital, Yonsei University College of Medicine, 50-1, Yonsei-ro, Seodaemun-gu, Seoul 03722, Korea; ohjaekeun@gmail.com; 4 Department of Neurosurgery, Hallym University Sacred Heart Hospital, 22, Gwanpyeong-ro 170 beon-gil, Dongan-gu, Anyang-si, Gyeonggi-do 14068, Korea; phlee@yuhs.ac; 5 Department of Neurology, Dongtan Sacred Heart Hospital, Hallym University College of Medicine, Hwaseong 18450, Korea

**Keywords:** cognitive status, degenerative spinal disease, geriatrics, MMSE

## Abstract

Cognitive status has been reported to affect the peri-operative and post-operative outcomes of certain surgical procedures. This prospective study investigated the effect of preoperative cognitive impairment on the postoperative course of elderly patients (*n* = 122, >65 years), following spine surgery for degenerative spinal disease. Data on demographic characteristics, medical history, and blood analysis results were collected. Preoperative cognition was assessed using the mini-mental state examination, and patients were divided into three groups: normal cognition, mild cognitive impairment, and moderate-to-severe cognitive impairment. Discharge destinations (*p* = 0.014) and postoperative cardiopulmonary complications (*p* = 0.037) significantly differed based on the cognitive status. Operation time (*p* = 0.049), white blood cell count (*p* = 0.022), platelet count (*p* = 0.013), the mini-mental state examination score (*p* = 0.033), and the Beck Depression Inventory score (*p* = 0.041) were significantly associated with the length of hospital stay. Our investigation demonstrated that improved understanding of preoperative cognitive status may be helpful in surgical decision-making and postoperative care of elderly patients with degenerative spinal disease.

## 1. Introduction

Cognitive status is one of the most important perioperative risk factors related to clinical outcomes in geriatric patients [1,2]. The elderly population has recently been increasing due to extended life expectancy, leading to an increase in the number of patients with degenerative spinal diseases and, thus, the number of spine surgeries. Therefore, spine surgeons are managing an increasing number of elderly people with spinal disorders and aging of the spine [3,4,5,6]. Moreover, these patients usually have an increased number of comorbidities, severe spinal degeneration, and reduced bone mineral density [7]. Furthermore, impaired cognitive status is common in elderly patients.

Previous studies have shown that cognitive impairment within the surgical population is associated with prolonged hospital stays, increased postoperative complications, and higher total medical cost [1,8]. However, preoperative cognitive status has been reported as one of the positive predictors of better outcomes after lumbar discectomy [9]. In addition, some authors have reported that cognitive-behavioral factors play an important role in postsurgical outcomes [10]. However, the effect of preoperative cognitive impairment on outcomes in elderly patients undergoing spine surgery for degenerative spinal disease has not yet been reported. Furthermore, the lack of accurate knowledge on the impact of preoperative cognitive status on the course of patients undergoing spine surgery restricts surgeons from planning adequate therapeutic strategies and providing appropriate postoperative care. Therefore, the aim of this study was to investigate the relationship between preoperative cognitive status and postoperative clinical outcomes in geriatric patients undergoing surgery for degenerative lumbar disease.

## 2. Materials and Methods

### 2.1. Patient Enrollment

**Figure 1:**
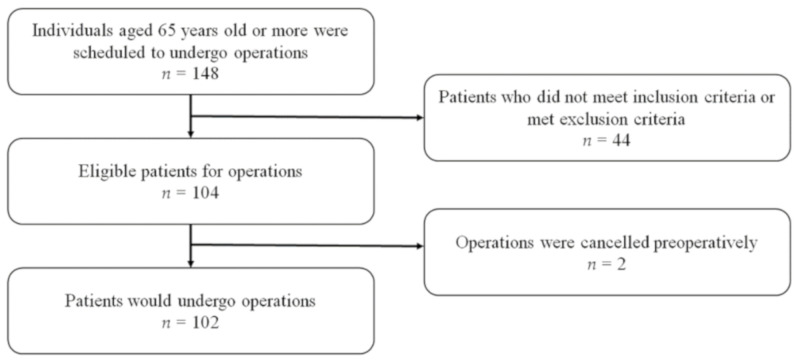
Participant enrollment. Exclusion from the study was based on the following criteria: (I) patients did not meet inclusion criteria (*n* = 44); (II) surgeries were cancelled before operations (*n* = 2).

Patients older than 65 years who underwent spine surgery for degenerative lumbar conditions between October 2015 and July 2016 at a single institution were enrolled in this study (Figure 1). The investigative protocol was approved by our hospital’s Institutional Review Board (NCT 02550626), and informed consent was obtained from all patients.

Among the 148 patients who underwent lumbar surgeries, we selected and prospectively studied 102 patients (68.9%) who met the inclusion criteria. Of these, 44 patients did not meet the inclusion criteria, and surgery was postponed for two patients. The inclusion criteria included the following: (I) patients aged ≥ 65 years who were scheduled to undergo spine surgeries, including discectomy, laminectomy, and fusion; (II) hospital stay of more than 3 days; and (III) indication for surgery including herniated nucleus pulposus, degenerative disc disease, spondylolisthesis, and spinal stenosis. We excluded patients with conditions that could affect general health such as spinal tumor-like lesions, infection, or trauma. Patients with known brain conditions, including cerebral contusions and Alzheimer’s disease, all of which can cause disorientation, were also excluded.

### 2.2. Perioperative Patient Assessment

Cognitive status of patients was evaluated using the Korean version of the mini-mental state examination (K-MMSE) [11]. The MMSE is one of the most widely used screening tests in clinical trials and in general practice for the detection of cognitive impairment in older adults [12]. In addition, it is easy to implement and has good test-retest reliability (0.80–0.95) [12,13,14]. The K-MMSE tests five cognitive functional areas: orientation, registration, attention-calculation, recall, and language. The maximum score is 30, with higher scores indicating better cognition. The patients were divided into three groups according to the K-MMSE score. Mild cognitive impairment (MCI) was defined as an MMSE score between 21 and 26 points, while moderate-to-severe cognitive impairment (MSCI) was defined as an MMSE score of ≤20 points. Normal cognition (NC) was defined as an MMSE score of ≥27 points. Baseline examinations were performed preoperatively to assess the postoperative clinical prognosis. These examinations were performed by two independent, trained research assistants who did not participate in the surgical care of the patients to reduce potential subjective bias. Patients were not evaluated on the day of surgery because of the potential confounding influence of intraoperative anesthetic medications.

Patient characteristics, MMSE scores, comorbidities, number of medications, and preoperative laboratory findings were examined to identify potential associations with preoperative cognitive status. Patient education level was assessed, and non-educated patients were assigned a score of 0; other patients were assigned points according to each additional year of education. We estimated patient degree of depression using the Beck Depression Inventory (BDI) [15], one of the most widely used psychometric tests for measuring the severity of depression. This inventory comprises a 0 to 63 rating system, and the higher the score, the higher the severity of depression. We also collected data about clinical outcomes related to postoperative prognosis. The data included the following: intraoperative estimated blood loss (EBL), admission to intensive care unit (ICU) after surgery, re-admission and revision, and presence of postoperative delirium. These factors were examined for identifying potential associations with preoperative cognitive status.

### 2.3. Statistical Analysis

Statistical analysis was performed with SPSS version 19 for Windows (IBM, Armonk, NY, USA). Data are presented as the mean ± standard deviation (SD). A Student’s t test, χ^2^ test, univariate linear regression analysis, and multivariate linear regression analysis with an enter method were used for statistical analysis. All variables with a significance level of *p* < 0.05 in the univariate analysis were included as independent variables in a forward stepwise regression method in the multivariate analysis. A value of *p* < 0.05 was considered statistically significant.

## 3. Results

### 3.1. Patient Data

Following study recruitment criteria, 102 patients who underwent spine surgeries for degenerative disease were enrolled in this study. The baseline characteristics of these 102 patients are presented in Table 1. At enrollment, 54 (52.9%) patients showed NC, 43 (42.2%) showed MCI, and five (4.9%) showed MSCI. There were no significant differences between the three groups in terms of demographic data, medical history, surgical method, or preoperative laboratory findings.

### 3.2. Outcome Data

The three study groups based on cognitive function (NC, MCI, and MSCI) did not differ significantly in terms of median length of stay, admission to ICU after surgery, total medical cost, or mean medical cost per day (Table 2). However, discharge destinations were significantly different in the three groups according to MMSE score (*p* = 0.014). The mean EBL was not significantly different in the three groups: 540 ± 454 mL in the MSCI group, 609 ± 521 mL in the MCI group, and 563 ± 641 mL in the NC group (*p* = 0.762). In addition, rates of re-admission, revision, and postoperative overall complications were not significantly different among the three groups. However, a significant difference was seen in cardiopulmonary complications among the three groups based on the MMSE score (*p* = 0.037).

Table 3 shows the results of the univariate and multivariate linear regression analyses for length of stay. From the univariate linear regression model, covariates with *p* < 0.2 (EBL, operation time (OT), hemoglobin (Hb) count, WBC count, platelet count, MMSE score, and BDI score) were included in the multivariate linear regression model. After the enter method analysis, OT (coefficient: 0.244, 95% confidence interval (CI) (0.000, 0.032), *p* = 0.049), WBC count (coefficient: −0.229, 95% CI (−0.001, 0.000), *p* = 0.022), platelet count (coefficient: 0.250, 95% CI (0.005, 0.043), *p* = 0.013), MMSE score (coefficient: −0.196, 95% CI (−0.763, −0.032), *p* = 0.033), and BDI score (coefficient: 0.190, 95% CI (0.005, 0.238), *p* = 0.041) were found to be significantly associated with length of stay.

## 4. Discussion

Cognitive decline is associated with a higher prevalence of several chronic medical conditions and may necessitate higher rates of procedures and surgeries [16,17]. Moreover, preoperative cognitive status has also been linked to the incidence of other adverse outcomes, including postoperative complications, longer hospital stays, and functional decline [16,18]. Robinson et al. reported that baseline cognitive impairment in older adults undergoing major elective surgery was related to adverse postoperative outcomes, including increased complications, prolonged hospital stays, and long-term mortality. [19]. Nonetheless, studies on the association between preoperative cognitive status and postoperative outcomes in adults who undergo surgery for degenerative spinal conditions are limited. As the frequency of spine surgery is increasing in parallel with the aging population and spinal degenerative diseases, a more comprehensive understanding of the relationship between preoperative cognitive status and clinical outcomes is important for predicting general prognosis and determining treatment plans following the spine surgery. In this prospective study, we discovered that discharge destination and OT were significantly different in patients with different preoperative cognitive status. Additionally, MMSE score, an indicator of cognitive status, was independently associated with the length of hospital stay in elderly patients following lumbar surgery.

In this analysis, we compared three groups of patients divided according to the preoperative cognitive status (NC, MCI, and MSCI as per the K-MMSE) who underwent elective surgery, and the three groups had significantly different discharge destinations (*p* = 0.014). Although these results were not statistically quantitatively correlated, we found that the better the preoperative cognitive status, the more likely it was for the patient to be discharged home (60% for MSCI vs. 91% for MCI vs. 96% for NC). This was not a surprising outcome because individuals with cognitive impairment would be less likely to function independently after surgery. Furthermore, patients with cognitive impairment are at a high risk of hospitalization due to a range of issues including the presence of complex medical illnesses, poor ability to manage chronic diseases, poor medication compliance, higher medication adverse effects, and lack of required social support [20,21]. Adogwa et al. reported that patients undergoing spine surgery for deformity with preoperative cognitive impairment were four times more likely to be discharged to a facility compared with patients with normal cognitive status [22]. Robinson et al. showed that geriatric patients with impaired cognition had a higher rate of discharge to institutions following surgery [19]. Furthermore, Nazir et al. suggested that discharge destination should be included in any model that seeks to predict hospitalization or rehospitalization risk for cognitively impaired individuals [23]. In addition, Capua et al. showed that the factors affecting the discharge destination after elective anterior cervical discectomy and fusion were older age (over 65 years), poorer patient functional status, OT over 4 h, and more severe American Society of Anesthesiology (ASA) class [24]. Since the preoperative cognitive status affects the discharge destination, it may be helpful for surgeons to consider this to plan the postoperative patient care.

We found the overall postoperative complications were not significantly different in the three MMSE groups, except for the cardiopulmonary complication (*p* = 0.037). Two of five patients in the MSCI group who underwent simple laminectomy developed complications of pneumonia and stable angina, respectively. Viramontes et al. demonstrated that there were strong associations between patients with pre-cognitive impairments undergoing total hip arthroplasty and increased hospital complications such as aspiration pneumonia [25].

Length-of-hospital stay (LOS) is a complex and multifaceted parameter with numerous measurable and intangible factors [26]. Some of the significant predictors of LOS are age, preoperative hemoglobin level, comorbidities, ASA score, type of surgery, fluid balance, volume of fluid transfused, postoperative pain intensity, dependency score, and postoperative complications [27,28,29,30]. However, some of these variables were omitted from the final model because of their modest effect and collinearity with other variables. Although previous studies have indicated that age was associated with LOS after surgery [31,32], age did not have any effect in our LOS model. In the present study, the linear regression models, including univariate and multivariate analyses, revealed that OT and LOS were positively correlated. This finding is consistent with the study of Kudo et al., which demonstrated that patients with long surgery time were more likely to have complications and subsequently have longer LOS [33]. In addition, our study showed that WBC count and platelet count were also associated with LOS. Some previous studies reported that low preoperative hemoglobin levels are associated with long LOS, but in our present study, including the linear regression models, WBC and thrombocyte counts, instead of hemoglobin levels, were associated with LOS [34,35]. In addition, Lakomkin et al. reported that platelet count was a significant predictor of postoperative complications following posterolateral lumbar fusion [35]. Although we did not directly compare the association between platelet count and occurrence of postoperative complications [34], patients who developed postoperative complications required longer hospitalization. Moreover, MMSE score and BDI score had significant effects on LOS in this study. Our results showed that as the MMSE score decreases and BDI score increases, the probability of that patient requiring a longer hospital stay increases. Some prior studies have demonstrated that a cut-off value of 24 points on the MMSE was predictive of a longer hospital stay [36]. MMSE scores tend to be lower with increasing age, and the decreased cognitive function leads to longer LOS [36]. Regarding depression, our findings are similar to those of the previous studies showing that higher the degree of depression [37], poorer the postoperative prognosis, including pain control, which might affect LOS.

Several factors contribute to the cost of spinal surgery: the specific type of spine surgery performed; the number of levels; comorbidities; and surgeon’s choices, such as implant selection [38]. Previous studies demonstrated that various patient factors, such as age, sex, race, insurance status, severity of illness, and length of stay, were also associated with the cost of lumbar spine surgery [39]. In our study, the linear regression models, including univariate and multivariate analyses, revealed that OT was the only variable associated with total medical cost. The longer the OT, the greater was the usage of drugs or complex procedures, which resulted in increased medical costs.

Although some studies comparing the cost of spine surgery have been performed [40,41,42], they did not particularly focus on the specific drivers of hospitalization cost or the prediction of its magnitude. However, while this was not investigated in our study, it was meaningful to identify the factors that affect the cost of medical care in terms of the clinical aspects of spine surgery. Estimation of the hospitalization cost for each patient undergoing spine surgery and the identification of the modifiable drivers of cost could allow physicians to understand the economic aspects of spine surgery and improve their clinical practice. Although the influence was not statistically significant, preoperative cognitive status measured by the MMSE is a possible factor that influences total medical cost in degenerative spine surgery.

Our study had some limitations that must be acknowledged. First, because of the small sample size, especially the number of patients in the MSCI group, and the fact that patients were recruited from a single institution in Korea, the generalizability of our findings may be limited, especially for patients with cultural differences and those with degeneration and higher BMI, such as in the United States or Europe. In the future, multicenter well-designed randomized studies with large samples may help further identify the risk factors associated with preoperative cognitive status in degenerative lumbar surgery. Second, this study was also limited by our inability to completely adjust for characteristics—known and unknown—that may have influenced the results. Although biases were reduced by controlling for significant variables via multivariate analysis, they could not be eliminated. Third, we did not investigate functional parameters, such as physical and social activity before and after surgery in the elderly patients who underwent surgery; we have considered including these parameters in future studies.

## 5. Conclusions

In conclusion, the role of preoperative cognitive impairment in elderly patients with degenerative spinal disease has not yet been elucidated. However, our data suggest that preoperative cognitive status is associated with discharge destination in such patients. Preoperative cognitive status, as measured by the MMSE, is one of the risk factors for LOS. Improved understanding of baseline cognition before surgery can help surgical decision-making, prediction of outcomes, and planning of postoperative care in elderly degenerative spine patients.

## Figures and Tables

**Table 1:** Baseline characteristics.

Characteristic	0 < MMSE < 20	21 ≤ MMSE ≤ 26	MMSE ≥ 27	*p*-Value
Demographic data				
Number of patients	5	43	54	-
Age (years)	73.6 ± 3.3	72.3 ± 4.7	70.9 ± 4.7	0.323
Sex; male, *n* (%)	0	13	21	0.083
Graduate	1.20 ± 2.68	6.40 ± 4.22	9.83 ± 4.07	0.424
Medical history				
Height (cm)	152.6 ± 2.8	156.1 ± 8.1	159.4 ± 8.6	0.083
Weight (kg)	53.6 ± 12.0	60.2 ± 7.5	62.2 ± 9.4	0.208
BMI (kg/m^2^)	22.9 ± 4.4	24.8 ± 2.8	24.4 ± 2.8	0.332
Number of medications	5.8 ± 2.8	5.5 ± 3.3	4.8 ± 3.1	0.866
HTN	4 (80%)	28 (65.1%)	35 (64.8%)	0.787
DM	0 (0%)	12 (27.9%)	11 (20.4%)	0.315
Cardiovascular disease	1 (20.0%)	9 (20.9%)	13 (24.1%)	0.925
Cerebrovascular disease	0 (0%)	6 (14.0%)	2 (3.7%)	0.140
Parkinson’s disease	1 (20.0%)	4 (9.3%)	8 (14.8%)	0.637
NP related disease	1 (20.0%)	3 (7.0%)	9 (16.7%)	0.321
BDI score	9.40 ± 4.83	14.58 ± 8.06	14.32 ± 8.68	0.329
Surgical method				
Spinal fusion, *n*	2	29	34	0.489
Decompression, *n*	3	14	20	
Laboratory findings				
Hemoglobin	13.2 ± 1.7	13.3 ± 1.3	13.8 ± 1.4	0.931
WBC	6.77 k ± 0.71 k	7.28 k ± 1.64 k	6.84 k ± 1.80 k	0.111
PLT	212.6 k ± 33.2 k	233.3 k ± 50.3 k	233.7 k ± 64.8 k	0.091
BUN	17.0 ± 2.9	17.1 ± 6.2	16.7 ± 4.1	0.064
Creatinine	0.65 ± 0.11	0.78 ± 0.22	0.78 ± 0.19	0.160
Albumin	3.9 ± 0.3	4.1 ± 0.3	4.2 ± 0.35	0.764

Abbreviations: BMI, body mass index; HTN, hypertension; DM, diabetes mellitus; NP, neuropsychiatric; BDI, Beck Depression Inventory; WBC, white blood cell; PLT, platelet; BUN, blood urea nitrogen.

**Table 2:** Outcome data.

	0 < MMSE < 20	21 ≤ MMSE ≤ 26	MMSE ≥ 27	*p*-Value
Number of patients	5	43	54	
Length of stay	10.6 ± 4.9	11.2 ± 5.8	9.4 ± 5.4	0.488
Admission to ICU after surgery, *n* (%)	1 (20.0%)	7 (16.3%)	5 (9.3%)	0.520
Discharged to home, *n* (%)	3 (60%)	39 (91%)	52 (96.3%)	0.014
Total medical costs ($)	7483.2 ± 2529.2	8644.9 ± 3446.8	7319 ± 3403.4	0.944
Mean medical cost per day ($)	765.3 ± 287.5	830.7 ± 271.6	814.4 ± 260.3	0.837
EBL (mL)	540 ± 454	609 ± 521	563 ± 641	0.762
OT (minutes)	186.2 ± 25.0	197.3 ± 67.6	192.3 ± 97.9	0.937
Re-admissions, *n* (%)	2 (40.0%)	11 (25.6%)	14 (25.9%)	0.796
Revision, *n* (%)	0 (0%)	7 (16.3%)	4 (7.4%)	0.273
Overall complications, *n* (%)	2 (40.0%)	16 (37.2%)	14 (25.9%)	0.450
Cardiopulmonary, *n* (%)	2 (40.0%)	1 (2.3%)	2 (3.7%)	0.037
Stroke, *n* (%)	0 (0%)	0 (0%)	1 (1.0%)	0.527
Wound infection, *n* (%)	0 (0%)	1 (1.3%)	2 (3.7%)	0.794
Postoperative pain, *n* (%)	0 (0%)	2 (4.7%)	1 (1.9%)	0.628
ASD, *n* (%)	0 (0%)	2 (4.7%)	4 (7.4%)	0.624
Postoperative delirium, *n* (%)	0 (0%)	10 (23.3%)	5 (9.3%)	0.098

Abbreviations: ICU, intensive care unit; EBL, estimated blood loss; OT, operation time; ASD, adjacent segment disease.

**Table 3:** Univariate and multivariate linear regression analysis of length of stay and other continuous variables.

Variables	Univariate Analysis				Multivariate Analysis			
	β-Coefficients	95.0% CI		*p*-Value	β-Coefficients	95.0% CI		*p*-Value
		Lower Bound	Upper Bound			Lower Bound	Upper Bound	
Age	−0.049	−0.292	0.176	0.623				
EBL	0.312	0.001	0.005	0.001	0.046	−0.002	0.003	0.716
OT	0.321	0.009	0.034	0.001	0.244	0.000	0.032	0.049
WBC	−0.136	−0.001	−0.000	0.174	−0.229	−0.001	0.000	0.022
Hb	−0.147	−10.411	0.200	0.139	−0.031	−0.912	0.657	0.748
PLT	0.241	0.005	0.042	0.015	0.250	0.005	0.043	0.013
MMSE	−0.169	−0.737	0.055	0.090	−0.196	−0.763	−0.032	0.033
BDI	0.240	0.030	0.277	0.015	0.190	0.005	0.238	0.041

Abbreviations: EBL, estimated blood loss; OT, operation time; WBC, white blood cell; Hb, hemoglobin; PLT, platelet; MMSE, mini-mental state examination; BDI, Beck Depression Inventory.

## Data Availability

Data available on request due to restrictions eg privacy or ethical. The data presented in this study are available on request from the corresponding author.
